# Risk factor assessment for clinical malaria among forest-goers in a pre-elimination setting in Phu Yen Province, Vietnam

**DOI:** 10.1186/s12936-019-3068-4

**Published:** 2019-12-20

**Authors:** Sara E. Canavati, Gerard C. Kelly, Cesia E. Quintero, Thuan Huu Vo, Long Khanh Tran, Colin Ohrt, Thang Duc Ngo, Duong Thanh Tran, Nicholas J. Martin

**Affiliations:** 1 Vysnova Partners, Inc., 4915 St. Elmo Ave., Bethesda, 20814 USA; 2 National Institute of Malariology, Parasitology and Entomology, 35 Trung Van, Hanoi, Vietnam; 3 Naval Medical Research Unit TWO, PSA Sembawang Deptford Rd., Building 7-4, Singapore, 759657 Singapore

**Keywords:** Forest-goers, Risk behaviors, Risk factor analysis, Malaria, Case–control study, Mobile and migrant populations, Malaria elimination, Vietnam

## Abstract

**Background:**

The transition from malaria control to elimination requires understanding and targeting interventions among high-risk populations. In Vietnam, forest-goers are often difficult to test, treat and follow-up for malaria because they are highly mobile. If undiagnosed, forest-goers can maintain parasite reservoirs and contribute to ongoing malaria transmission.

**Methods:**

A case–control study was conducted to identify malaria risk factors associated with forest-goers in three communes in Phu Yen Province, Vietnam. Cases (n = 81) were residents from the study area diagnosed with malaria and known to frequent forest areas. Controls (n = 94) were randomly selected forest-going residents from within the study area with no identified malaria infection. Participants were interviewed face-to-face using a standard questionnaire to identify malaria risk factors. Logistic regression was used to calculate odds ratios (ORs) and 95% CI for risk factors after adjusting for socio-demographic characteristics.

**Results:**

Among the cases, malaria infection varied by species: 66.7% were positive for *Plasmodium falciparum*, 29.6% for *Plasmodium vivax*, and 3.7% were diagnosed as mixed infection. Cases were less likely than controls to use treated nets (aOR = 0.31; 95% CI 0.12–0.80), work after dark (aOR = 2.93; 95% CI 1.35, 6.34), bath in a stream after dark (aOR = 2.44; 95% CI 1.02–5.88), and collect water after dark (aOR = 1.99; 95% CI 1.02–3.90).

**Conclusions:**

As Vietnam moves toward malaria elimination, these findings can inform behaviour change communication and malaria prevention strategies, incorporating the risk of after-dark and water-related activities, in this priority and difficult-to-access population group.

## Background

The malaria burden in Vietnam has been reduced under the Millennium Development Goals and the national malaria programme is now transitioning away from control operations with an aim to eliminate the disease by 2030 [1, 2]. As malaria transmission declines, countries pursuing an elimination agenda must address increased heterogeneity in the distribution of remaining pockets of malaria infection [3, 4]. Heterogeneity exists both in a geographic context, relating to the spatial distribution of transmission, and in a demographic context, where specific population groups may be at a higher risk of malaria infection or serve as a significant asymptomatic parasite reservoir. To disrupt and halt the potential for ongoing transmission, malaria programmes must implement effective strategies that actively seek out, detect and treat all malaria infections, especially in hard to reach groups [5–7].

In the Greater Mekong Subregion (GMS), epidemiological complexities such as the presence of mobile and migrant populations including forest-goers who mostly reside in remote locations, and the spread of anti-malarial drug resistance pose key challenges for malaria programmes [8, 9]. To address these concerns, Vietnam’s elimination programme includes improving access to early diagnosis and prompt effective treatment, ensuring uniform intervention coverage for at-risk populations, and strengthening the malaria epidemiological surveillance system and capacity to implement robust malaria epidemic response [1]. However, even in Vietnam, forest-goers maybe not reached or covered by health services, due in part to their remote location and mobile lifestyles. These characteristics are challenges to effectively test and treat these populations for malaria, as well as track and implement routine malaria prevention measures [10]. If undiagnosed, forest-goers can maintain parasite reservoirs and contribute to ongoing transmission. Due to these factors surveillance systems are not optimized to target these hard to reach groups in Vietnam and the magnitude of this problem is not yet fully elucidated.

To target interventions towards high-risk populations, it is essential for programmes to effectively analyse and identify the associated risk of malaria [11, 12]. In malaria elimination settings, the identification of risk factors becomes difficult using standard methods due to low numbers of infections, calling for novel surveillance approaches [7, 13]. In support of Vietnam’s pursuit of malaria elimination, a case–control study was conducted to identify associations between risk behaviours and malaria infection status among forest-goers in Phu Yen Province, Vietnam.

## Methods

### Setting and study area

**Fig. 1:**
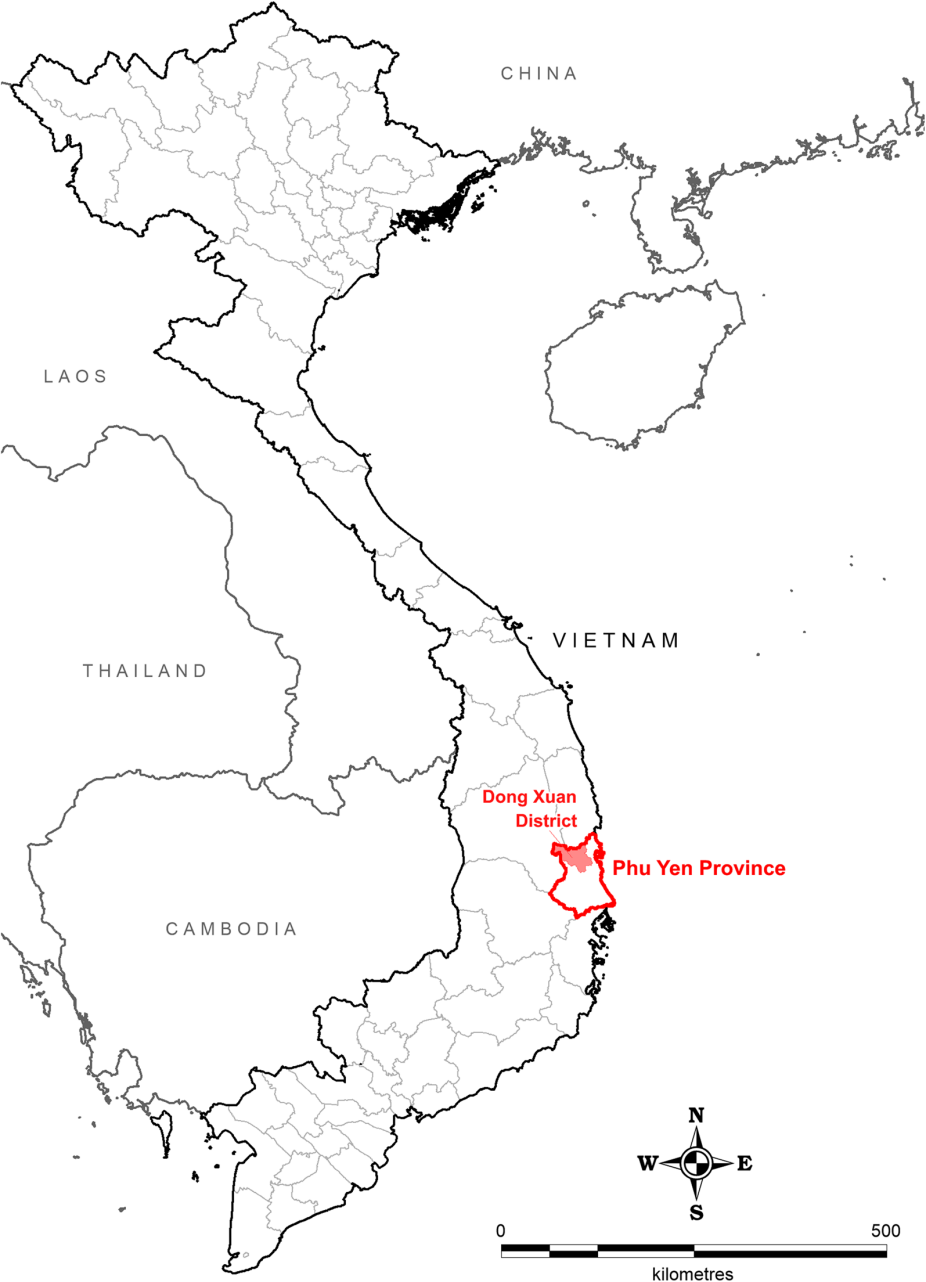
Dong Xuan District, Phu Yen Province, Vietnam study location map

**Fig. 2:**
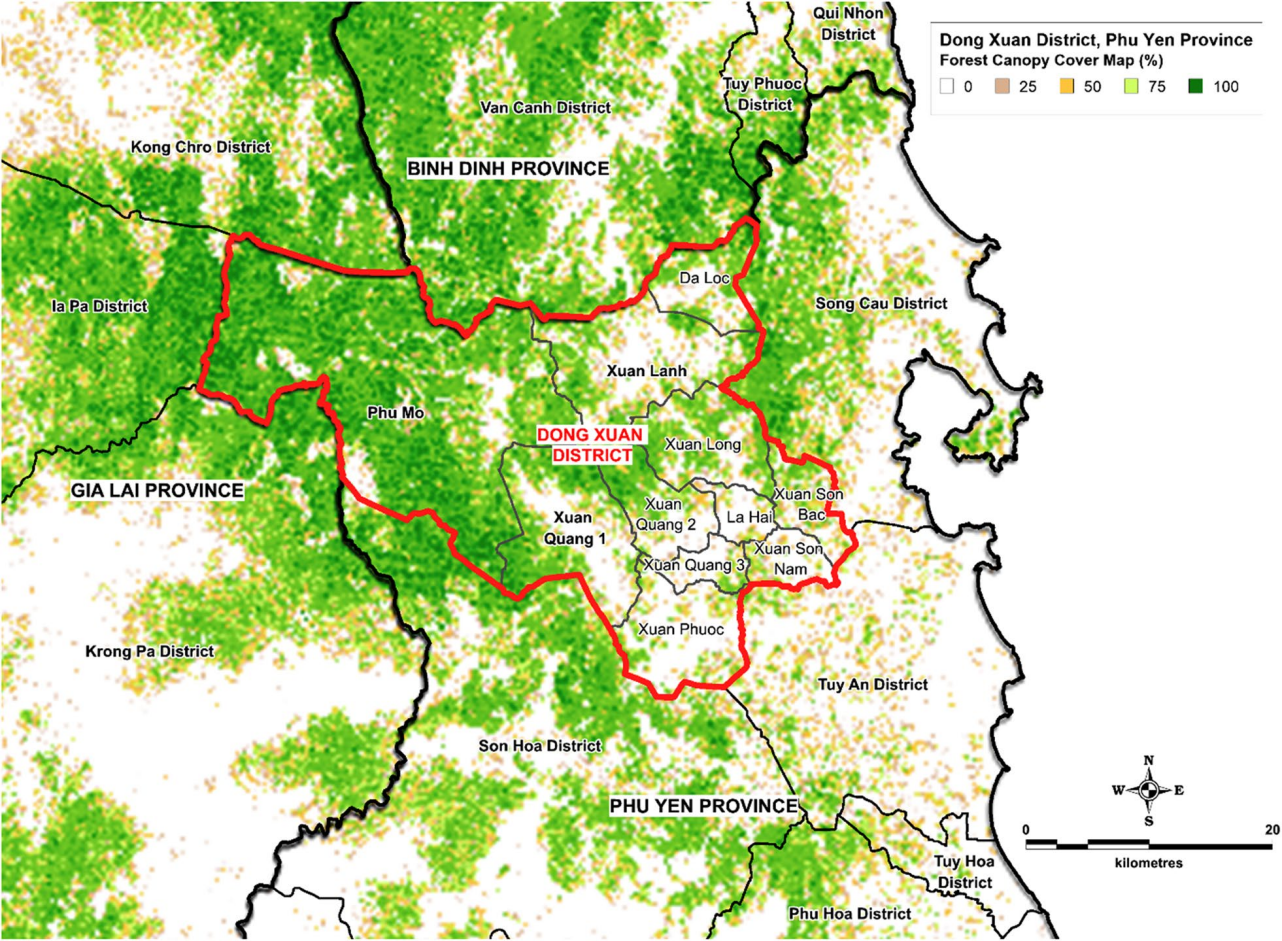
Study commune location sites overlaid on forest tree cover canopy percentage data (Tree cover canopy percentage spatial layer adapted from 30 m resolution global land cover satellite imagery spatial modelling data [47])

The study was conducted in Phu Mo, Xuan Lanh, and Xuan Quang 1 communes in Dong Xuan district, northwest Phu Yen Province, located in South-Central Vietnam (Fig. 1). These study sites were selected by the Vietnam National Institute of Malariology, Parasitology and Entomology due to the high malaria burden, a large number of forest-fringe settlements, and their location in bordering two Tier 1 Artemisinin Resistant provinces as defined by the World Health Organization Global Plan for Artemisinin Resistance Containment [14]. The three study communes are the western communes of Dong Xuan District, located directly adjacent to the mountainous and densely forested central highlands region of Vietnam (Fig. 2). Elevation varies steeply within the study area ranging from approximately 50 metres above sea level in the valleys to more than 1300 metres above sea level in the western mountainous regions. Malaria transmission in Phu Yen occurs primarily from May through November with bimodal peaks in transmission following the rainy seasons [15, 16]. In 2016, the study area had an approximate population of 18,000 [17]. Due to the characteristics of the province’s resources as well as the structure of the occupation, a significant proportion of the study area working population is known to frequently travel to, and sleep in, the forest for their livelihoods [16].

### Study design

A population-based case–control study, matched at the village level, was conducted in the study site from September to November 2016.

The sample size for this case–control study was calculated using the below formula [18].

$${\text{n}} = \frac{{\left\{ {z_{1 - \alpha /2} \sqrt {2{\text{P}}_{2}^{*} \left( {1 - {\text{P}}_{2}^{*} } \right)} + z_{1 - \beta } \sqrt {{\text{P}}_{1}^{*} \left( {1 - {\text{P}}_{1}^{*} } \right) + {\text{P}}_{2}^{*} \left( {1 - {\text{P}}_{2}^{*} } \right)} } \right\}^{2} }}{{\left( {{\text{P}}_{1}^{*} - {\text{P}}_{2}^{*} } \right)^{2} }}$$

The sample size was calculated by assuming an expected ratio of 3, the prevalence of 0.63 for control group did not exposure to the study risk factor bases on the previous study [19] and case to control ratio of 1:1. With the power of test of 80% at the significant level of 5%, the sample size needed was 81 cases and 81 controls.

A case was considered as any resident within the three study site communes who met the following criteria: (1) visit to their local commune health facility due to suspected malaria; (2) a positive test for malaria by rapid diagnostic test (RDT; SD BIOLINE Malaria Ag P.f/P.v), and microscopy [20]; (3) willingness and availability to participate in the study; and (4) self-reported history of sleeping overnight in the forest. Those who were not eligible for the study met one or more of the following exclusion criteria: (1) a negative test for malaria by RDT or microscopy; (2) prior malaria diagnosis within the preceding month; (3) taking malaria prophylaxis or treatment in the preceding 2 weeks; (4) clinically diagnosed cases without any diagnostic test for confirmation; or (5) unwillingness or unavailability to participate in the study.

Controls were enrolled within a 2-week time period following identification of the case. As a means to minimize selection bias, controls that were absent were revisited twice before an alternative eligible household member or neighbor was selected. Inclusion criteria for population controls were defined as (1) a negative malaria test by RDT; (2) willingness and availability to participate in the study; (3) resident within the same study site village of the case; and (4) self-reported history of sleeping overnight in the forest. Criteria excluding controls included: (1) a positive test for malaria by RDT or microscopy, (2) taking malaria prophylaxis or treatment in the preceding 2 weeks; and (3) unwillingness or unavailability to participate in the study. Controls were randomly selected from the case’s home village. All eligible patients that were diagnosed with malaria in the three selected communes during the study period were invited to participate in the study.

Written informed consent was obtained from all study participants or their legal guardian. All study participants were informed before the start of the interview about the project’s goals, the topic and type of questions, the intended use of results for scientific publications, and their right to refuse the interview, interrupt the conversation at any time, and withdraw all given information during or after the interview, without any negative consequences. Anonymity was guaranteed and the confidentiality of interviewees assured by assigning a unique code number to each participant. Local authorities and community leaders were informed of the purpose and expected the duration of the study.

### Data collection and procedures

Cases and controls were interviewed face-to-face using a structured questionnaire (Additional file 1)  to capture key socio-demographic data. Data were also collected on behaviour in the forest, including seasonal exposure, sleeping habits, use of hammocks, after-dark activities; and knowledge of and attitudes towards malaria prevention measures. More than one answer could have been accepted per participant, and the answers were categorized accordingly. A standardized questionnaire was developed in English with input from Vietnam National Institute of Malariology, Parasitology and Entomology (NIMPE) staff. It was then translated into Vietnamese, and back-translated into English by a fluent bilingual health expert prior to pre-testing. All members of the study team were trained in standardized interviewing techniques for consistent data collection. Data were captured at the household of participants using smartphones and KLL collect (Kathmandu Living Labs, Nepal) data collection software.

Logic checks, range limits and required answering were programmed into the digital data collection forms as a means to minimize data collection errors. Data were uploaded into an online data storage application (Ona Systems, Nairobi, Kenya) at the point of collection. Study supervisors reviewed and uploaded data daily to ensure data quality, completeness and consistency. Data were stored on encrypted via 256 RSA SSL servers with ISO 27001 certification. Answers to the study questionnaires and disease status of participants were kept confidential.

### Data analysis

Data were analysed using R software (Epi and BMA packages). Chi-square and t-test for normally distributed variables were used to assess statistically significant differences in socio-demographic characteristics between the cases and controls. Social economic status (SES) was defined by quintile technique using principal components and factor analysis of asset score developed from nine asset items, including variables such as electricity, water resources, ownership of motorbike, television, radio, mobile phone, household assets [21]. Crude odds ratios (cORs) and its 95% confidence intervals (CI) were calculated for risk behaviours of forest-goers between cases and controls by a univariate analysis. The variables that were known to be associated with malaria status from previous studies (including ethnicity, gender, age, education, and SES [22]) were considered in the multivariate logistic regression analysis to calculate the adjusted odd ratios (aORs) for risk behaviours of forest-goers between cases and controls. A 0.05 level of significance was applied for all statistical analysis conducted.

## Results

A total of 90 cases were identified and screened from confirmed malaria cases at the three commune health clinics and 96 neighborhood controls within the study area, which exceeded target enrollment to allow for losses following review of inclusion/exclusion criteria. Out of 186 participants, 11 (nine cases and two controls) were excluded from the study as they did not meet the inclusion criteria. In total 81 cases and 94 controls were enrolled in the study, resulting in an approximate 1:1 case to control ratio. Among the cases, malaria infection varied by species: 66.7% were positive for *Plasmodium falciparum*, 29.6% for *Plasmodium vivax*, and 3.7% were diagnosed as mixed infection.

### Sociodemographic factors

Of the 175 enrolled participants, 154 (88.0%) were male and 147 (84.0%) had high economic status. Participants were from three ethnic groups: Kinh (34.9%), Cham (54.9%) and Bana (10.3%). Mean age was 36.2 ± 11.8 years (Table 1). There were no significant difference with regard to socio-demographic characteristics between cases and controls (p > 005), with the exception of age. The mean age of cases (34.2 ± 11.7) was significantly lower than that of controls (37.8 ± 11.6). The distribution in gender between cases and controls was not significantly different, males comprised about 88% of the population for each group. The two highest SES group accounted for a large proportion of the cases (39.5%) and controls (43.6%) in this study population.

**Table 1:** Socio-demographic characteristics of cases and controls in Dong Xuan district, Phu Yen province, Vietnam

Characteristics	Cases (n = 81) (%)	Controls (n = 94) (%)	Total (n = 175) (%)	p-value
Age (mean-SD)	34.2 ± 11.7	37.8 ± 11.6	36.2 ± 11.8	0.04
Gender				0.90
Male	71 (87.7%)	83 (88.3%)	154 (88.0%)	
Female	10 (12.3%)	11 (11.7%)	21 (12.0%)	
Education level				0.54
Illiterate	18 (22.2%)	28 (29.8%)	46 (26.3%)	
Primary school	25 (30.9%)	21 (22.3%)	46 (26.3%)	
Secondary	16 (19.8%)	19 (20.2%)	35 (20.0%)	
High school and above	22 (27.2%)	26 (27.7%)	48 (27.4%)	
Ethnicity				0.70
Kinh	28 (34.6%)	33 (35.1%)	61 (34.9%)	
Cham	43 (53.1%)	53 (56.4%)	96 (54.9%)	
Bana	10 (12.3%)	8 (8.5%)	18 (10.3%)	
Household economic status^a^				0.895
Highest	17 (21%)	18 (19.1%)	35 (20%)	
High	15 (18.5%)	23 (24.5%)	38 (21.7%)	
Medium	18 (22.2%)	17 (18.2%)	35 (20%)	
Low	17 (21%)	18 (19.1%)	35 (20%)	
Lowest	14 (17.3%)	18 (19.1%)	32 (18.3%)	
Main work in the forest				0.21
Aloe seeking and hunting	16 (19.8%)	25 (26.6%)	41 (23.4%)	
Tree planting	43 (53.1%)	50 (53.2%)	93 (53.2%)	
Trapping	6 (7.4%)	10 (10.6%)	16 (9.1%)	
Other^b^	16 (19.8%)	9 (9.6%)	25 (14.3%)	

^a^By Quintile

^b^Making charcoal and logging

Among main types of work in the forest, tree planting was not significantly different between cases and controls (53.1%); however, aloe seeking/hunting (19.8% compared to 26.6%) and trapping (7.4% compared to 10.6%) were lower in cases than in controls. The proportion of forest-goers who had graduated from secondary school or above was not significantly different between the cases and controls; however, self-reported illiteracy was lower among cases (22.2%) compared with controls (29.8%). Similarly, the proportion of those from the Kinh ethnic group was not significantly different between cases (34.6%) and control (35.1%), but the proportion of Cham and Bana varied between the cases (53.1% and 12.3%) and controls (56.4% and 8.5%), respectively (Table 1). Cham ethnic group accounted for more than 80% of illiterate respondents in this study population (p < 0.001) (Table 2).

**Table 2:** Education levels of cases and controls by ethnic group

Ethnicity	Illiterate (n = 46) (%)	Primary (n = 46) (%)	Secondary (n = 35) (%)	Highschool (n = 48) (%)	Total (n = 175) (%)	p-value
Bana	6 (13.0%)	8 (17.4%)	3 (8.6%)	1 (2.1%)	18 (10.2%)	
Cham	38 (82.6%)	33 (71.7%)	12 (34.3%)	13 (27.1%)	96 (54.9%)	< 0.001
Kinh	2 (4.4%)	5 (10.9%)	20 (57.1%)	34 (70.8%)	61 (34.9%)	

### Risk factors for malaria

Risk factors evaluated in this study are presented in Table 3 and were grouped into four general categories: seasonal exposure, sleeping habits, use of nets (including hammock nets), and after-dark activities. Of the risk behaviours investigated in the study, five were significantly associated with malaria. These included: use of treated nets (protective), sleeping in a hut without walls, and participating in after dark activities including collecting water, bathing in streams, and working.

**Table 3:** Univariate and multivariate analysis of risk factors for malaria

Risk factors	Cases (n = 81)	Controls (n = 94)	cOR (95% CI)	aOR (95% CI)
Seasonal exposure				
Number of nights slept in the forest per year (mean-SD)	130.8 ± 80.8	118.5 ± 70.4	1.00 (1.00–1.01)	1.00 (0.99–1.00)
Go to the forest during high risk months (October–November and December)	60 (45.5%)	72 (54.5%)	0.87 (0.44–1.74)	0.76 (0.36–1.60)
Sleeping habits (in hammock-bed or floor)				
Sleep without any kind of net*^#^	1 (50%)	1 (50%)		
Use treated nets*	7 (24.1%)	22 (75.9%)	0.31 (0.12–0.77)	0.31 (0.12–0.80)
Use untreated nets*	78 (47.3%)	87 (52.7%)	2.09 (0.52–8.37)	1.89 (0.44–8.09)
Use net (treated or untreated) for the whole night				
Never or sometimes	13 (54.2%)	11 (45.8%)	(ref)	(ref)
Usually or always	68 (45%)	83 (55%)	0.69 (0.29–1.65)	0.62 (0.24–1.63)
Slept in a hut without walls^#^	15 (79%)	4 (21%)		
Sleep in hammock with an untreated net*	33 (43.4%)	43 (56.6%)	0.82 (0.45–1.49)	0.94 (0.48–1.84)
After dark activities				
Collecting water after dark	57 (52.8%)	51 (47.2%)	2.00 (1.07–3.75)	1.99 (1.02–3.90)
Bathing in the stream after dark	72 (50.4%)	71 (49.6%)	2.59 (1.12–5.99)	2.44 (1.02–5.88)
Work after dark	26 (65%)	14 (35%)	2.70 (1.30–5.63)	2.93 (1.35–6.34)

*Multiple response possible

^#^OR not computed due to insufficent number of observations

Sleeping with treated nets was associated with significantly lower odds of being a malaria case; the crude OR was 0.31 (95% CI 0.12–0.77) and did not change after adjusting for age, gender, ethnicity, education level and SES (aOR: 0.31; 95% CI 0.12–0.80). Using an untreated net or sleeping without any kind of net increased the odds of being a malaria case, however, these association was not statistical significant after adjustment, with the 95% CI range of 0.44 to 8.09.

The association between sleeping without any kind of net and the odds of being a malaria case was not significant (cOR 1.16; 95% CI 0.07–18.88). After adjusting for age, gender, ethnicity, education levels and SES the aOR was 2.21 (95% CI 0.12–41.08).

There was a significant association between sleeping without any kind of net, sleeping in a hut without walls and the odds of being a malaria case. However, the OR was not computed due to a low number of observations (n = 4).

There was an association between after dark activities and being a malaria case. Collecting water after dark doubled the odds of being a malaria case, with no confounding by demographic factors over the association identified (cOR = 2.00, 95% CI 1.07–3.75; aOR = 1.99, 95% CI 1.02–3.90). Bathing in a stream after dark increased the odds of being a malaria case (cOR = 2.59, 95% CI 1.12–5.99; aOR = 2.44, 95% CI 1.02–5.88). Similarly, there was strong association between work after dark and the odds of being a malaria case, people who worked after dark were significantly more likely to be a malaria case compared to who did not work after dark (cOR = 2.70, 95% CI 1.30–5.63; aOR = 2.93, 95% CI 1.35–6.34).

## Discussion

While the results from this case–control study are largely consistent with similar studies regarding forest-goers and their risk of malaria in the GMS [23–26], this study reveals additional findings, particularly in relation to after dark behaviour for forest goers residing and working in central Vietnam. The ability to validate findings from other studies in the GMS allows for generalization of regional approaches and can provide contrast to approaches and challenges seen in Africa. Given the epidemiological variances and complexities unique to the GMS in comparison to African settings related to the challenges of forest malaria and vector biology, region specific studies of this nature are imperative to inform malaria control and elimination strategies. Additionally, this study allows for a better understanding of demographic characteristics and risk factors specific to forest-goers in Phu Yen province, which should be taken into account when designing interventions at the province level.

Of particular note, and contrary to previous findings [26–29] in Vietnam, neither going to the forest during high-risk months (OR = 0.87), nor sleeping more nights in the forest per year (OR = 1.00), were associated with increased malaria infection in this study population. This suggests that other factors, such as after-dark behaviour, in this setting may have more influence on risk of malaria among forest-goers. Expanded epidemiological research is required within the GMS to better understand risk factors associated with seasonal exposure and duration of stay in the forest. Consistent with other studies in the GMS there were significantly more male than female forest-going respondents [24, 30, 31], and these data support current knowledge and practices that males should be the primary target for malaria prevention and control efforts among forest-goers.

Overall, there were no statistically significant differences in demographics between the cases and controls in this study with the exception of age. Larger comparative case–control studies are necessary to better understand why age was a risk factor in this study population. Age has also been found to be a risk factor in certain demographics, such as the Jarai ethnic group along the Cambodia-Vietnam border, with young men, in particular, tending to forego certain protective behaviours, such as using bed nets [24]. Should a perceived lack of importance of personal protection or complacency be identified among high-risk groups, such as young forest-going males, it may be necessary to develop alternative messages such as the potential impact of an individual’s behaviour, even when asymptomatic, may have on vulnerable groups such as infants, pregnant women and the elderly within their extended communities. Given the heterogeneity of culture among ethnic groups, stratifying such studies by ethnic group could also provide additional insight for tailoring malaria elimination activities for high risk populations.

Respondents who reported sleeping in a hut without walls were five time more likely to contract malaria than those who slept in huts with walls. However, the relatively small number of respondents who reported sleeping in a hut without walls (less than 11%) limit the interpretation of this finding. Though the number of total participants from this study was small, several recent publications have also highlighted the significant impact sleeping structures have on malaria incidence [32–34]. In a recent systematic review and meta-analysis, residents of ‘modern’ houses were observed to have a 47% lower odds of malaria infection and a 45–65% lower odds of clinical malaria, compared to residents of ‘traditional’ houses in settings across Asia, Africa and Latin America [35]. The building body of evidence, including data from this study (OR = 5.1 for sleeping in a hut without walls), suggests improved and enclosed sleeping structures should be an area to target future interventions [32–42]. This presents clear challenges in the context of mobile and migrant forest-going populations and novel approaches are clearly required to adequately address such a risk in these settings.

Most participants in the study reported using some kind of mosquito net. However, one of the most striking results from this study is that only 8.6% of cases and 23.4% of controls reported using treated nets, despite the protective advantages associated with using treated nets. Further research is needed to determine whether the predominant use of untreated nets is due to access, perceived advantages of untreated nets (such as net type, comfort, portability, suitability, durability, or aesthetics), or cultural barriers. Further attention should also be directed towards the suitability and acceptability of insecticide-treated hammock net options given the proportion of participants using untreated hammock nets in this study and external research findings in this area in relation to their potential protective benefits to forest-going individuals [43, 44].

Although after-dark activities including collecting water, bathing and working were carried out by respondents in both groups, cases were significantly more likely to engage in these practices than controls. Correspondingly their risk of infection was more than double that of controls. Further attention should be given to determine the factors attributable to why cases engaged in after-dark activities such as awareness of risk, cultural influences, environmental, and/or occupational practices and constraints. For example, if a specific forest-based occupation workday typically ends after dusk or is located far from a water source, those individuals might have no choice but to carry out essential activities after dark.

The study identified that all participants in both categories belonged to one of three ethnic groups. As reflected in Table 2, there is a strong correlation between ethnic group and literacy levels, with the Cham accounting for more than 80% of self-report illiterate respondents. Ethnic group risk analysis data can be used to inform future studies with regard to ethnic groups and their association with specific forest occupations, as well as educational and economic status. Targeted strategies including awareness and behaviour change communication (BCC) interventions should obviously also consider these factors during design and delivery. This is particularly crucial given the reducing malaria burden in the GMS will require increasingly targeted and specialized interventions to support elimination objectives.

Employers in Vietnam could also serve a role to integrate study findings into malaria control and prevention practices. It is difficult to identify and target mobile individuals working for short periods of time, however, working with their static employers may prove less challenging. Whilst it is known there are populations of illegal forest workers in Vietnam, a significant proportion of high-risk forest going groups are also engaged in formal employment within forest settings, such as logging, forest plantations and other agriculture work. Previous studies have shown that employers in the GMS understand the financial benefit in keeping their workforce malaria-free, thus may be motivated to implement malaria prevention practices [45]. Identifying these employers could help address issues related to uptake and use of treated nets and sleeping in an enclosed structure, both factors associated with lower risk of malaria infection in this study.

Matching cases with neighbourhood controls minimized bias related to variations in geographical accessibility to a health facility, few statistically significant differences between the cases and controls were found. This speaks to the challenges of conducting research in elimination settings with low incidence. Although the design did not allow for investigation of ecological risk factors, it did provide statistical power to evaluate micro-epidemiological features related to individual and household factors, as well as an efficient method of data collection.

Due to the nature of the study, data relied on self-reporting; results may, therefore, have been subject to both recall and interviewer bias. Since there was no blinding in this study, it is possible participants altered their answers to be more socially desirable or to produce what they felt was the expected outcome. The fact that cases demonstrated health-seeking behaviour by presenting to the clinic, might suggest a differential bias. It is, therefore, possible that there was a misclassification of the outcome. The study also did not determine whether the two groups were exposed to the same level of malaria BCC and whether they had comparable access to bed nets. These factors could have biased the results. Misclassification of malaria status of controls could have also been an issue, as they were screened with RDTs, which have decreased sensitivity for the detection of low blood parasite concentrations observed in asymptomatic malaria [46]. Those with low blood parasite concentrations could have been misclassified as free from malaria. This study was also unable to capture those who seek services in the private sector; the proportion of forest-goers in the study area who present to the private sector is unknown. Furthermore, those who were selected as controls may have contracted malaria and been treated by the private sector. In such cases, they would not appear in the public records as malaria patients and would, therefore, have been considered eligible as a control.

## Conclusions

This case–control study provides valuable information on malaria risk factors of forest going individuals in the Dong Xuan district of Vietnam. The case–control method utilized in this study was a cost-effective and flexible approach to study the heterogeneous malaria risk in an area with diminishing, but persistent malaria burden. Further qualitative studies or larger sample size survey would help determine whether barriers to prevention practices affected cases more than controls and if this is related to factors such as the type of forest work. Findings from this study provide a basis to inform future research on forest-goers in Vietnam to ascertain the extent to which access, age, knowledge or attitude impact the engagement of high-risk behaviours. Given that the use of treated nets was found to be protective and sleeping in a hut without walls was a significant risk factor to contract malaria, further investigation of these risk factors is warranted. With age being the only significant demographic difference, future studies can help determine if younger groups engage in riskier behaviours. Furthermore, these findings can inform future interventions to optimally target, at-risk and heterogeneous populations in malaria elimination settings. Data on risk factors and knowledge can be integrated into national initiatives aimed to combat malaria, both within the public and private sector. This study yields valuable results on risk factors in the Dong Xuan district of Vietnam, both in regards to differences, as well as commonalities, of case and control individuals.

## Supplementary information

**Additional file 1.** Structured questionaire used during face-to-face interviews.

## Data Availability

The datasets generated during and/or analysed during the current study are available from the corresponding author on reasonable request.

## Abbreviations

BCC: behaviour change communication
CI: confidence interval
GMS: Greater Mekong Sub-region
NIMPE: Vietnam National Institute of Malariology, Parasitology and Entomology
OR: odd ratio
cOR: crude odd ratio
aOR: adjusted odd ratio
RDT: rapid diagnostic tests
SES: social economic status
