# Supplementary material for: Risk factor assessment for clinical malaria among forest-goers in a pre-elimination setting in Phu Yen Province, Vietnam
Source: Malar J. 2019 Dec 20;18:435. doi: 10.1186/s12936-019-3068-4 (PMC6923829; doi:10.1186/s12936-019-3068-4)
Supplement: Supplementary file 1 — Additional file 1. Structured questionaire used during face-to-face interviews. [file 12936_2019_3068_MOESM1_ESM.pdf]

Long_HH_for_QT_23Sep15_V1

1. Mã số hộ, nếu không có sử dụng tên của chủ hộ

Household number if available, if not, house owner

____

2. Họ tên người trả lời (Hỏi chủ hộ, nếu không có nhà hỏi người có kinh nghiệm <60t - Nhóm Không mắc Sốt rét có yếu tố nguy cơ giống BN mắc SR: PV BN mắc SR ở rừng/rẫy thì PV nhóm đối chứng là người sống gần nhà có đi rừng/rẫy; BN SR phỏng vấn mắc SR ở Nhà thì PV người sống gần nhà BN không đi rừng/rẫy (Viết tắt chữ cái đầu của họ đệm và tên) vd: Nguyễn Văn Anh (viết N.V.A)

Interviewee initials - In non-malaria Patient should have same type of risk factors: if interviewed malaria patient got malaria in forest/farm near fringe forest - interviewed control person should be forest goers/farm goer; if malaria patient got malaria at home so interviewed control person should be nearby household - not forest/farm goer (HH head, if not senior person available person under 60

____

3. Dân tộc

Ethnicity

- ◯ Kinh
- ◯ Ê-đê
- ◯ Ba-na
- ◯ Chăm
- ◯ Vân Kiều
- ◯ Tà-ôi
- ◯ Pa-co
- ◯ Khác

4. Năm sinh

Year of birth

____

5. Giới tính
Gender

- ◯ Nam
- ◯ Nữ

6. Trình độ học vấn cao nhất trong hộ gia đình là gì?
Highest level of education in the HH

- ◯ Không biết chữ
- ◯ Tiểu học
- ◯ Trung học cở sở
- ◯ Trung học phổ thông
- ◯ Trên trung học phổ thông

7. Gia đình ông/bà sống ở đây bao năm rồi?
How many years does your family live here?

____

8. Gia đình ông/bà có ai sang Lào không?
Do Your family go to Lao?

- ◯ Có
- ◯ Không

9. Mỗi năm gia đình ông/bà sang Lào bao nhiêu lần?
How many times do your family go to Lao per year?

____

10. Gia đình ông/bà sang Lào để làm gì?
What do your family do in Lao?

____

. NHỮNG CÂU HỎI VỀ KIẾN THỨC VÀ THÁI ĐỘ

11. Ông/bà biết gì về nguyên nhân gây bệnh sốt rét? (để người trả lời tự nói nếu không nói được ý nào thì hỏi lại ý đó)
Do you know anything about the causes of malaria (spontaneous first and then ask)

- [ ] a. Muỗi sốt rét đốt.
- [ ] b. Rừng và bìa rừng là nơi có nhiều muỗi sốt rét
- [ ] c. Khi hỏi đồng ý với: Muỗi sốt rét chỉ đốt từ chập tối đến rạng sáng.
- [ ] d. Khi hỏi đồng ý với: Muỗi sốt rét đốt người thường không thấy đau, không thấy ngứa nhưng truyền bệnh rất nguy hiểm
- [ ] e. Khi hỏi đồng ý với: Sốt rét sẽ truyền cho người thân nếu không chữa trị kịp thời
- [ ] f. Khi hỏi đồng ý với: Rừng và bìa rừng là nơi có nhiều muỗi sốt rét
- [ ] g. Không biết

12. Theo ông/bà có những cách nào phòng tránh sốt rét/bảo vệ để không bị muỗi đốt?(để người trả lời tự nói nếu không nói được ý nào thì hỏi lại ý đó)?

How to prevent malaria/ not to be infected by mosquitoes ?

- [ ] a. Luôn luôn ngủ mùng/võng mùng khi ở nhà, khi đi rừng và khi đi rẫy
- [ ] b. Đi lấy nước và tắm ở sông/suối TRƯỚC khi trời sẩm tối
- [ ] c. Bôi kem chống muỗi lúc chập tối trước khi đi ngủ (cho các hoạt động vào buổi tối và ban đêm: săn bắn, uống rượu, xem tivi, hẹn hò, tắm, ăn tối,...)
- [ ] d. Nhớ bôi kem chống muỗi và giăng mùng trước khi uống rượu
- [ ] e. Mang mùng/võng mùng đi tẩm hóa chất khi được cán bộ y tế yêu cầu
- [ ] f. Khi có sốt phải đến ngay cơ sở y tế để xét nghiệm, chuẩn đoán và điều trị
- [ ] g. Khi hỏi đồng ý với: Luôn luôn ngủ mùng/võng mùng khi ở nhà, khi đi rừng và khi đi rẫy
- [ ] h. Khi hỏi đồng ý với: Đi lấy nước và tắm ở sông/suối TRƯỚC khi trời sẩm tối
- [ ] j. Khi hỏi đồng ý với: Bôi kem chống muỗi lúc chập tối trước khi đi ngủ (cho các hoạt động vào buổi tối và ban đêm: săn bắn, uống rượu, xem tivi, hẹn hò, tắm, ăn tối,...)
- [ ] k. Khi hỏi đồng ý với: Nhớ bôi kem chống muỗi và giăng mùng trước khi uống rượu
- [ ] l. Khi hỏi đồng ý với: Mang mùng/võng mùng đi tẩm hóa chất khi được cán bộ y tế yêu cầu
- [ ] m. Khi hỏi đồng ý với: Khi có sốt phải đến ngay cơ sở y tế để xét nghiệm, chuẩn đoán và điều trị
- [ ] n. Không biết

13. Theo ông/bà cảm thấy bệnh sốt rét nguy hiểm ở mức nào? (Từ 1 là Không nguy hiểm gì? Đến 5 là rất nguy hiểm/có thể gây chết người)

How dangerous is malaria? From level 1 to level 5 (very dangerous)

- ◯ Mức 1 (Không nguy hiểm)
- ◯ Mức 2
- ◯ Mức 3
- ◯ Mức 4
- ◯ Mức 5 (Rất nguy hiểm/có thể chết người)

14. Gia đình ông/bà có bao nhiêu người? (Những người ăn và ngủ ở nhà ông/bà 6 tháng trở lại đây)

How many people in your household ? (People ate and sleeped in last 6 months in this house)

____

15. Nhà ông/bà có người nhập cư (người từ nơi khác/tỉnh khác đến) ở không?

Any migrants in this house ?

- ◯ Có
- ◯ Không

16. Có bao nhiêu người mắc sốt rét trong 5 năm gần đây của gia đình ông/bà? (cả gia đình)

How many family members had malaria in last 5 years ?

____

17. Số lần mắc sốt rét trong 5 năm gần đây của gia đình ông/bà? (cả gia đình)

Total times had malaria in last 5 years

____

18. Số lần ông/bà mắc sốt rét trong 5 năm gần đây? (cá nhân, nếu không ghi số 0)

How many times have you had malaria in last 5 years ? (if interviewee has not had malaria, please write 0)

____

19. Lần mắc sốt rét gần đây nhất của ông/bà cách đây bao nhiêu THÁNG? (Nếu tháng nay ghi 0)

19. Lần mắc sốt rét gần đây nhất của ông/bà cách đây bao nhiêu THÁNG? (Nếu tháng này ghi 0)

How long from your most recent malaria ? (by Month)

____

20. Ông/bà có biết nơi nào có thuốc sốt rét ngoài các cơ sở y tế nhà nước? (ngoài bệnh viện, trung tâm y tế và trạm y tế nhà nước)

Do you know where you can take anti malaria drug?

- ◯ Có
- ◯ Không

21. Đó là ở đâu?

Where did you get it?

- ☐ Phòng khám/bệnh nhân tư nhân ở Việt Nam
- ☐ Nhà thuốc tư nhân ở Việt Nam
- ☐ Nơi khác ở Việt Nam
- ☐ Phòng khám/bệnh nhân tư nhân tại Lào
- ☐ Nhà thuốc tư nhân tại Lào
- ☐ Nơi khác ở Lào

22. Ông/bà có vào rừng để làm việc không?

Have you ever go to forest to work?

- ◯ Có
- ◯ Không

. NHÓM CÓ ĐI RỪNG

23. Ông/bà có ngủ ở trong rừng không - ít nhất một đêm trong năm qua? (Nếu người trả lời không ngủ lại vẫn hỏi các câu liên quan đến hoạt động trong rừng trừ câu hỏi liên quan đến ngủ và mùng)

Do you sleep in the forest? (if no, still ask some questions about activities in forest)

- ◯ Có
- ◯ Không

» Hoạt động trong rừng

24. Công việc chính (nghề chiếm nhiều thời gian nhất) của ông/bà trong rừng là gì?
What is your main work in the forest ?

- ◯ Trồng cây
- ◯ Làm than củi
- ◯ Khai thác gỗ
- ◯ Tìm trầm
- ◯ Thợ săn
- ◯ Bẫy thú
- ◯ Làm rẫy
- ◯ Công nhân
- ◯ Thợ hồ
- ◯ Khai thác quặng
- ◯ Buôn bán
- ◯ Việc khác

25. Mô tả chi tiết công việc chính của ông/bà? Làm rõ những công việc cụ thể khi làm việc đó là gì? (VD: Trồng cây gì?, săn con gì? Bẫy con gì?Loại gỗ khai thác là gì)
Most important question on the list this end the multiple choice to determine future malaria tranmission risk classification to who to give prophylaxis and hammock nets; e.g. exact type of planation or wood exploitation or hunter or trapper - improve list with local people)

____

26. Ông/bà có làm việc sau khi trời tối hoặc trước khi trời sáng?
Do you work after dark or before dawn?

- ◯ Có
- ◯ Không

27. Tên công ty/tên chủ đang làm việc? (nếu có)
Company/Ower Name (if you are hired)

____

28. Số người làm tại nơi đó?
Numberworkersat_site

____

29. Ở trong rừng_Tính trung bình, thời gian Ông/bà ngủ (làm việc cho nhóm về trong ngày) trong chòi/lều này khoảng bao ngày trong một năm? (Ngày)
In average, how many days you sleep in forest per year ?

____

30. Loại rừng Ông/bà đã đi vào? (Chọn 1 hoặc cả hai)
Type of forest you go to? (Choices one or both)

- ☐ Rừng già/rừng sâu/Rừng nguyên sinh
- ☐ Bìa rừng (Rừng tái sinh/Rừng trồng)

31. Nhóm của ông/bà ngủ nơi đâu?
Where your work group sleep?

- ☐ Nhà
- ☐ Lều có tường che chắn có thể phun thuốc
- ☐ Lều bạt
- ☐ Võng có mùng
- ☐ Võng không mùng
- ☐ Trên mặt đất
- ☐ Khác

32. Tháng nào thì ông/bà vào rừng làm việc này?

Select which months you spend sleeping in the forest with this work

- [ ] Tháng 1
- [ ] Tháng 2
- [ ] Tháng 3
- [ ] Tháng 4
- [ ] Tháng 5
- [ ] Tháng 6
- [ ] Tháng 7
- [ ] Tháng 8
- [ ] Tháng 9
- [ ] Tháng 10
- [ ] Tháng 11
- [ ] Tháng 12

33. Khi làm việc ở trong rừng, nơi ngủ của ông/bà có bao nhiêu người NGỦ lại ít nhất là 1 đêm trong năm qua (có thể làm thuê)? (tính luôn người được hỏi)

How many people sleep with you at the working location (may be hired labour) (include interviewee)

____

34. Trong những người ngủ này, có bao nhiều người mắc sốt rét trong vòng 2 năm trở lại đây? (tính luôn người được hỏi)

In those people sleeping with interviwee, how many people get malaria in recent 2 years (include interviewee)

____

35. [illegible]

35. Ông/bà biết bao nhiêu người/ca mắc sốt rét trong nơi làm việc trong vòng 2 năm qua?
How many malaria cases do you aware of in the area around in the last 2 yrs?

____

36. Có KHOẢNG bao nhiêu người ngủ trong bán kính 2km xung quang nơi làm việc của ông/bà ở trong rừng trong 2 năm trở lại đây?
How many people sleep forest in the 2km area in 2yrs? (APPROXIMATELY)

____

37. Từ chỗ ngủ hoặc nơi làm việc trong rừng tới chỗ có sóng điện thoại ĐI BỘ mất KHOẢNG bao nhiêu PHÚT - Ghi bằng "0" nếu có sóng?
Howmany minutestonearestcellphone_coverage by walking from sleeping area (record "0" if there is signal) (APPROXIMATELY)

____

38. Khi ngủ ở RỪNG thường mấy giờ ông/bà ngủ?
What time do you sleep at FOREST?

--:-- --
____

39. Ông/bà có dùng mùng hay võng khi ngủ rừng không?
Do you use net/hammock when sleep?

- ○ Có
- ○ Không

» » . NHÓM ĐI RỪNG có NGỦ MÙNG/VÕNG MÙNG

40. Nếu đo lường mức độ dùng mùng/võng CẢ ĐÊM thì nhóm của ông/bà dùng ở mức độ nào? (đọc đáp án)
How often do members of your sleepinggroup sleep under net/hammock net in the forest?

- ○ Không bao giờ
- ○ Thỉnh thoảng
- ○ Thường xuyên
- ○ Luôn luôn

41. Có tổng cộng bao nhiêu mùng và võng tại nơi ngủ của nhóm của ông/bà?
Total net and hammock do sleeping group have in sleeping area in forest

____

42. Loại mùng và võng ở đó là gì? (CHỌN TẤT CẢ CÁC LOẠI MÀ HỌ CÓ)
Types of nets and hammocks? (Select all of them)

- [ ] Mùng đơn
- [ ] Mùng đôi
- [ ] Mùng đơn tẩm thuốc MỀM
- [ ] Mùng đơn tẩm thuốc CỨNG
- [ ] Mùng đôi tẩm thuốc MỀM
- [ ] Mùng đôi tẩm thuốc CỨNG
- [ ] Mùng đơn tẩm thuốc tồn lưu MỀM
- [ ] Mùng đơn tẩm thuốc tồn lưu CỨNG
- [ ] Mùng đôi tẩm thuốc tồn lưu MỀM
- [ ] Mùng đôi tẩm thuốc tồn lưu CỨNG
- [ ] Võng không có mùng
- [ ] Võng có mùng không có khóa kéo
- [ ] Võng có mùng có khóa kéo
- [ ] Võng có mùng tẩm không có khóa kéo
- [ ] Võng có mùng tẩm có khóa kéo
- [ ] Võng có mùng tẩm tồn lưu không khóa kéo
- [ ] Võng có mùng tẩm tồn lưu có khóa kéo

- [ ] Võng không có mùng
- [ ] Võng có mùng không có khóa kéo
- [ ] Võng có mùng có khóa kéo
- [ ] Võng có mùng tẩm không có khóa kéo
- [ ] Võng có mùng tẩm có khóa kéo
- [ ] Võng có mùng tẩm tồn lưu không khóa kéo
- [ ] Võng có mùng tẩm tồn lưu có khóa kéo
- [ ] Khác

43. Nhóm của ông/bà có TỔNG CỘNG bao nhiêu MÙNG loại thường ở nơi ngủ trong rừng?
How many total regular nets for sleeping group do THEY have?

____

44. Trong đó, Có bao nhiêu cái MÙNG được tẩm hóa chất?
How many treated nets for sleeping group?

____

45. Có tổng cộng bao nhiêu cái võng tại nơi ngủ của nhóm ông/bà?
How many hammocks for sleeping group do they have?

____

46. Trong đó có bao nhiêu cái võng có mùng ở trong nhóm làm việc của ông/bà?
How many hammocks with NETs does sleeping group have?

____

47. Trong đó, có bao nhiêu cái võng có mùng được tẩm hóa chất?
How many treated hammocks do you have in sleeping group?

____

48. Ông/bà có muốn dùng mùng/võng mùng được tẩm CẢ ĐÊM khi ngủ ở trong rừng?
Do you want to use treated net/hammock when sleeping in the forest ?

- ( ) Có

Không

49. Loại mùng/võng nhóm ông/bà thích dùng NHẤT để ngủ - CẢ ĐÊM trong rừng là gì? (Cho ví dụ ông bà

Type of net/hammock your workgroup like to use the most? Show examples that we have purchased, plus program regular net and hammock

- Mùng đơn tẩm thuốc MỀM
- Mùng đơn tẩm thuốc CỨNG
- Mùng đôi tẩm thuốc MỀM
- Mùng đôi tẩm thuốc CỨNG
- Mùng đơn tẩm thuốc tồn lưu MỀM
- Mùng đơn tẩm thuốc tồn lưu CỨNG
- Mùng đôi tẩm thuốc tồn lưu MỀM
- Mùng đôi tẩm thuốc tồn lưu CỨNG
- Võng có mùng tẩm không có khóa kéo
- Võng có mùng tẩm có khóa kéo
- Võng có mùng tẩm tồn lưu không khóa kéo
- Võng có mùng tẩm tồn lưu có khóa kéo
- Võng có mùng tẩm không có khóa kéo
- Võng có mùng tẩm có khóa kéo
- Võng có mùng tẩm tồn lưu không khóa kéo
- Võng có mùng tẩm tồn lưu có khóa kéo
- [illegible]

◯ Khác

50. Mô tả mùng/võng đó để biết chính xác nó như thế nào?
Describle net/hammock your workgroup like the most

____

51. Dự án muốn tìm phương án để tất cả những người đi rừng có mùng tẩm thuốc. Vậy, gia đình ông/bà muốn dự án chia sẻ bao nhiêu tiền cho việc mua mùng/võng tầm thuốc?
We want to find a way for all forest goers to have a treated net. One way is to get more expensive nets and cost-share. How much money do you want us to share to buy hammock/net?

____

52. Một cách khác là sử dụng mùng có giá cả rẻ hơn. Vậy loại mùng nào sau đây ông/ bà có thể chấp nhận sử dụng?
Another way would be to provide less expensive nets. What other types can be acceptable that you would still want to use?

- ☐ Mùng đơn tẩm thuốc MỀM
- ☐ Mùng đơn tẩm thuốc CỨNG
- ☐ Mùng đôi tẩm thuốc MỀM
- ☐ Mùng đôi tẩm thuốc CỨNG
- ☐ Mùng đơn tẩm thuốc tồn lưu MỀM
- ☐ Mùng đơn tẩm thuốc tồn lưu CỨNG
- ☐ Mùng đôi tẩm thuốc tồn lưu MỀM
- ☐ Mùng đôi tẩm thuốc tồn lưu CỨNG
- ☐ Võng có mùng tẩm không có khóa kéo
- ☐ Võng có mùng tẩm có khóa kéo
- ☐ Võng có mùng tẩm tồn lưu không khóa kéo
- ☐ Võng có mùng tẩm tồn lưu có khóa kéo
- ☐ Võng có mùng tẩm không có khóa kéo
- ☐ Võng có mùng tẩm có khóa kéo
- ☐ Võng có mùng tẩm tồn lưu không khóa kéo
- ☐ Võng có mùng tẩm tồn lưu có khóa kéo
- ☐ Khác

53. Theo ông/bà, giải pháp phù hợp nhất mọi người sẽ sử dụng là gì?
In the interviewee opinion, what is the most affordable solution that people will use?

54. Ông/bà có thích loại mùng tồn lưu cứng của Chương trình Quốc gia cấp không? (giải pháp rẻ nhất là khoảng 85.000đ)
Would the double regular nets provided by National Program be an accptable solution? (cheapest solution $3.75)

- Có
- Không

55. Nếu như không thích, ông bà cho biết lý do tại sao?
If no, why not?

56. Ông/bà có thích loại võng có mùng mà chương trình quốc gia cấp?
Would the hammock nets provided by National Program be an acceptable solution?

- Có
- Không

57. Nếu như không thích, ông bà cho biết lý do tại sao?
If no, why not?

58. Ông/bà có loại mùng/võng nào dưới đây Ở NHÀ? Đọc đáp án
Do you have net and/or hammock at home? Reading choices

- Mùng
- Võng có mùng
- Võng không có mùng nhưng có mùng kèm theo hoặc bọc võng
- Không có cả ba

59. Ông/bà có thể mang những mùng/võng có mùng này vào rừng không?
Can you bring nets/hamock that you have from home to forest?

- Có
- Không

60. Nếu không mang Tại sao không?
If no, why not - consider list

- ☐ Không có do không tiền mua
- ☐ Không có do không biết chỗ mua
- ☐ Không có do xa quá không đi được để mua
- ☐ Nhà đang dùng không thể lấy đi
- ☐ Quá nặng để mang đi
- ☐ Khác

61. Ông/bà có muốn tẩm hóa chất cho mùng của ông/bà (chưa được tẩm) không?
Would you be willing to treat your nets?

- ○ Có
- ○ Không

62. Ông/bà có muốn tẩm hóa chất cho cả võng và mùng của võng ông/bà (chưa được tẩm) không?
Would you be willing to treat your entire nets/hammock ?

- ○ Có
- ○ Không

63. Nếu không tẩm cả võng và mùng của võng, thì chỉ tẩm mùng của võng được không?
If no, Would you be willing to treat just only the net of hammocks?

- ○ Có
- ○ Không

64. Theo đánh giá của cán bộ điều tra, có bao nhiêu màn cần được tẩm cho nhóm ngủ trong rừng này ?
In the interviewer's judgement, how many nets need to be treated for this work group?

65. Theo đánh giá của cán bộ điều tra, cần mua bao nhiêu mùng mới cho nhóm ngủ trong rừng này?
In the interviewer's judgement, how many nets need to be purchased?

____

66. Theo đánh giá của cán bộ điều tra, loại nào sau đây cần được mua mới?
In the interviewer's judgement, which types need to be purchased?

- ☐ Mùng đơn tẩm thuốc MỀM
- ☐ Mùng đơn tẩm thuốc CỨNG
- ☐ Mùng đôi tẩm thuốc MỀM
- ☐ Mùng đôi tẩm thuốc CỨNG
- ☐ Mùng đơn tẩm thuốc tồn lưu MỀM
- ☐ Mùng đơn tẩm thuốc tồn lưu CỨNG
- ☐ Mùng đôi tẩm thuốc tồn lưu MỀM
- ☐ Mùng đôi tẩm thuốc tồn lưu CỨNG
- ☐ Võng có mùng tẩm không có khóa kéo
- ☐ Võng có mùng tẩm có khóa kéo
- ☐ Võng có mùng tẩm tồn lưu không khóa kéo
- ☐ Võng có mùng tẩm tồn lưu có khóa kéo
- ☐ Võng có mùng tẩm không có khóa kéo
- ☐ Võng có mùng tẩm có khóa kéo
- ☐ Võng có mùng tẩm tồn lưu không khóa kéo
- ☐ Võng có mùng tẩm tồn lưu có khóa kéo
- ☐ Khác

67. Theo đánh giá của cán bộ điều tra, loại màn nào có giá cả hợp lý nhất và được chấp nhận nhiều nhất (số lượng và loại) cho nhóm ngủ trong rừng này? (Bao nhiêu cái và loại nào? Thêm giá vào mỗi cái mùng)

In the interviewer's judgement, what is the most affordable, acceptable solution for this sleeping group?(Number of nets and which type)? (Add cost of each net to the list)

____

68. Theo đánh giá của cán bộ điều tra, giải pháp nào được cho là hợp lý nhất để giảm trừ lây truyền sốt rét cho nhóm ngủ trong rừng này? (số mùng và loại mùng mà nhóm này thực sự sẽ dùng)

In the interviewer's judgement, what is the best solution to reduce malaria transmission for this sleeping group (Number of nets and which type that people will really use)?

____

69. Theo đánh giá của điều tra viên, Nếu loại màn cần mua có giá cao, nhóm ngủ trong rừng này có sẵn sàng chi trả 1/2 giá tiền với dự án không? (có thể hỏi người dân để trả lời câu hỏi này)

In the interviewer's judgement, If the chosen type of net has high price, will the people in this working group be willing and able to share half cost with project?

- ◯ Có
- ◯ Không

70. Ở trong rừng, lúc chập tối đến rạng sáng, ông/bà thường bị muỗi đốt khi đang làm gì - kể tất cả?

What do you do when mosquitoes bother you? (Select all)

- ☐ Đi tắm
- ☐ Ăn cơm
- ☐ Nấu cơm
- ☐ Đang làm việc
- ☐ Đi săn
- ☐ Ngồi nghỉ
- ☐ Ngủ
- ☐ Khi đi từ rừng/rẫy về nhà
- ☐ Muỗi không chích tôi

☐ Muỗi không chích tôi

71. Ở trong rừng_Làm gì thì bị muỗi cắn nhiều nhất
What do you do at the time when mosquitoes bother you most - Main

- ◯ Đi tắm
- ◯ Ăn cơm
- ◯ Nấu cơm
- ◯ Đang làm việc
- ◯ Đi săn
- ◯ Ngồi nghỉ
- ◯ Ngủ
- ◯ Khi đi từ rừng/rẫy về nhà
- ◯ Muỗi không chích tôi

74. Ở trong rừng, Ông/bà có đi lấy nước sinh hoạt/đi ra ngoài sau khi trời tối hoặc trước khi trời sáng không?
Is patient going to get drinking water outside location after dark or before dawn?

- ◯ Có
- ◯ Không

75. Ở trong rừng, Ông/bà có đi tắm ở suối/khe nước sau khi trời tối không?
Bathing outside house after dark

- ◯ Có

- Không

76. Công việc thứ hai của ông/bà tại rừng là gì? (Nếu có) (Nếu không có chuyển qua câu 76)
Other types of work in the forest (If no, move to question 76)

- [ ] Trồng cây
- [ ] Làm than củi
- [ ] Khai thác gỗ
- [ ] Tìm trầm
- [ ] Thợ săn
- [ ] Bẫy thú
- [ ] Làm rẫy
- [ ] Công nhân
- [ ] Thợ hồ
- [ ] Khai thác quặng
- [ ] Buôn bán
- [ ] Việc khác
- [ ] Không có

77. Mô tả chi tiết công việc thứ hai của ông/bà? Làm rõ những công việc cụ thể khi làm việc đó là gì?
Most important question on the list this end the multiple choice to determine future malaria tranmission risk classification to who to give prophylaxis and hammock nets; e.g. exact type of planation or wood exploitation or hunter or trapper - improve list with local people)

____

78. Ông/bà có làm việc sau khi trời tối hoặc trước khi trời sáng?
Do you work after dark or before dawn?

- Có
- Không

79. Tên công ty /tên chủ thứ 2 đang làm việc?
Company/Ower Name

____

80. Số người làm tại nơi đó?
Numberworkersat_site

____

80a. Trung bình trong một năm, có bao nhiều ngày ông/bà về nhà sau khi trời tối và trước khi trời sáng?
In average, How many days do you walk back from forest after dark before dawn

____

81. Ông/bà có đi vào rẫy gần rừng không?
Do you go to farm near the forest (answer as above)

- Có
- Không

. NHÓM CÓ ĐI RẪY GẦN RỪNG

82. Ở trong rẫy Ông/bà có ngủ ở trong chòi/lều trên rẫy không - ít nhất là 1 đêm trong 1 năm qua? (Nếu người trả lời không ngủ lại vẫn hỏi các câu liên quan đến hoạt động trong rẫy trừ câu hỏi liên quan đến ngủ và mùng)
Did you sleep in the farm near forest fringe - at least 1 night in last year? (if no, still ask some questions about activities in farm)

- Có
- Không

» Hoạt động trong rẫy

83. Trong rẫy, ông/bà trồng cây gì/nuôi con gì (việc chính)?
Main work when you go to farm

- ◯ Trồng cây sắn
- ◯ Trồng cây lúa
- ◯ Trồng cây cà phê
- ◯ Làm than củi
- ◯ Khai thác gỗ
- ◯ Bẫy thú
- ◯ Làm rẫy
- ◯ Làm thuê
- ◯ Thợ hồ
- ◯ Việc khác

84. Mô tả chi tiết công việc thứ hai của ông/bà? Làm rõ những công việc cụ thể khi làm việc đó là gì?
Most important question on the list this end the multiple choice to determine future malaria tranmission risk classification to who to give prophylaxis and hammock nets; e.g. exact type of planation or wood exploitation or hunter or trapper - improve list with local people)

____

85. Số người làm tại nơi đó?
Numberworkersat_farm

____

86. Ở trong RẪY GẦN RỪNG_Tính trung bình, thời gian Ông/bà ngủ (hoặc làm việc trong rừng) trong chòi/lều này khoảng bao ngày trong một năm? (Ngày)
In average, how many days you sleep in farm per year ?

____

87. Nhóm của ông/bà ngủ nơi đâu?
Where are your sleepinggroup sleep?

- [ ] Nhà
- [ ] Lều có tường che chắn có thể phun thuốc
- [ ] Lều bạt
- [ ] Võng có mùng
- [ ] Võng không mùng
- [ ] Trên mặt đất
- [ ] Khác

88. Tháng nào thì ông/bà vào RẪY làm việc này?

Select which months per year you spend sleeping in the FF with this work

- [ ] Tháng 1
- [ ] Tháng 2
- [ ] Tháng 3
- [ ] Tháng 4
- [ ] Tháng 5
- [ ] Tháng 6
- [ ] Tháng 7
- [ ] Tháng 8
- [ ] Tháng 9
- [ ] Tháng 10
- [ ] Tháng 11
- [ ] Tháng 12

89. Khi làm việc ở trong RẪY, nơi ngủ của ông/bà có bao nhiêu người NGỦ lại ít nhất là 1 đêm trong năm qua (có thể làm thuê)? (Bao gồm cả người được hỏi)

How many people sleep with you at the sleeping-working location (denominator for nets)? (Include interviewee)

____

90. Trong những người ngủ này, có bao nhiều người mắc sốt rét trong vòng 2 năm trở lại đây? (Tính luôn người được hỏi)

In those people sleeping with interviwee, how many people get malaria 2 years (include interviewee)

____

91. [illegible]

91. Tại nơi làm việc của ông/bà ở trong rẫy, có KHOẢNG bao nhiêu người ngủ trong bán kính 0,5 km trong 2 năm trở lại đây ?

HowmanypeoplesleepinhalfKm in farm in 2 yrs (APPROXIMATELY)

____

92. Trong 2 năm qua, có KHOẢNG bao nhiêu người ông bà biết bị mắc sốt rét trong phạm vi 0.5 Km tại nơi làm việc ở trong rẫy?

Howmanymalariacases do you know in2yr_farm in half km (APPROXIMATELY)

____

93. Từ chỗ ngủ trong rẫy (hoặc chỗ làm việc với nhóm không ngủ lại) tới chỗ có sóng điện thoại ĐI BỘ mất bao nhiêu phút - Ghi bằng "0" nếu có sóng?

Howmany minutestonearestcellphone_coverage by walking (record "0" if there is signal)

____

94. Khi ngủ ở rẫy thường mấy giờ ông/bà ngủ?

what time do you sleep at farm?

--:-- --

____

95. Ông/bà có dùng mùng hay võng khi ngủ rẫy không?

Did you use net/hammock when sleep in FF?

- ◯ Có
- ◯ Không

» » . NHÓM ĐI RẪY CÓ NGỦ MÙNG/VÕNG MÙNG

96. Khi ở RẪY_nhóm của ông/bà có dùng mùng/võng HẾT CẢ ĐÊM ở mức nào? (đọc đáp án)

How often do members of your sleepinggroup under net/hammock net in the FF?

- ◯ Không bao giờ
- ◯ Thỉnh thoảng
- ◯ Thường xuyên
- ◯ Luôn luôn

97. Có tổng cộng bao nhiêu mùng và võng tại nơi ngủ của nhóm của ông/bà?

Total nets and hammocks do sleepinggroup have in sleeping in forest

____

98. Loại mùng và võng ở đó là gì? (CHỌN TẤT CẢ CÁC LOẠI MÀ HỌ CÓ)
Types of nets and hammocks (select all type they have)

- [ ] Mùng đơn
- [ ] Mùng đôi
- [ ] Mùng đơn tẩm thuốc MỀM
- [ ] Mùng đơn tẩm thuốc CỨNG
- [ ] Mùng đôi tẩm thuốc MỀM
- [ ] Mùng đôi tẩm thuốc CỨNG
- [ ] Mùng đơn tẩm thuốc tồn lưu MỀM
- [ ] Mùng đơn tẩm thuốc tồn lưu CỨNG
- [ ] Mùng đôi tẩm thuốc tồn lưu MỀM
- [ ] Mùng đôi tẩm thuốc tồn lưu CỨNG
- [ ] Võng không có mùng
- [ ] Võng có mùng không có khóa kéo
- [ ] Võng có mùng có khóa kéo
- [ ] Võng có mùng tẩm không có khóa kéo
- [ ] Võng có mùng tẩm có khóa kéo
- [ ] Võng có mùng tẩm tồn lưu không khóa kéo
- [ ] Võng có mùng tẩm tồn lưu có khóa kéo

- [ ] Võng không có mùng
- [ ] Võng có mùng không có khóa kéo
- [ ] Võng có mùng có khóa kéo
- [ ] Võng có mùng tẩm không có khóa kéo
- [ ] Võng có mùng tẩm có khóa kéo
- [ ] Võng có mùng tẩm tồn lưu không khóa kéo
- [ ] Võng có mùng tẩm tồn lưu có khóa kéo
- [ ] Khác

99. Nhóm của ông/bà có TỔNG SỐ bao nhiêu mùng ở nơi ngủ trong RẪY?
How many regular nets for sleepinggroup do you have?

____

100. Trong đó, Có bao nhiêu cái mùng được tẩm hóa chất?
How many regular nets treated for sleepinggroup?

____

101. Tổng cộng bao nhiêu cái võng tại nơi ngủ của nhóm ông/bà?
How many hammock for sleepinggroup do you have?

____

102. Trong đó, có bao nhiêu cái võng có mùng ở trong nhóm của ông/bà?
How many hammock with NET do sleepinggroup have?

____

103. Trong đó, có bao nhiêu cái võng mùng được tẩm hóa chất?
how many treated hammocks do you have in sleepinggroup?

____

104. Ở trong rẫy_Từ chòi/lều này tới rừng (bất kì) khoảng bao nhiêu Km? (Nếu trong rừng ghi số 0)
How far is your farm huts which is nearest to the forest from the farm?

____

105. Loại mùng nhóm ông/bà thích dùng NHẤT để ngủ CẢ ĐÊM trong RẪY là gì?
Type of net/hammock your workgroup like most to use

- Mùng đơn tẩm thuốc MỀM
- Mùng đơn tẩm thuốc CỨNG
- Mùng đôi tẩm thuốc MỀM
- Mùng đôi tẩm thuốc CỨNG
- Mùng đơn tẩm thuốc tồn lưu MỀM
- Mùng đơn tẩm thuốc tồn lưu CỨNG
- Mùng đôi tẩm thuốc tồn lưu MỀM
- Mùng đôi tẩm thuốc tồn lưu CỨNG
- Võng có mùng tẩm không có khóa kéo
- Võng có mùng tẩm có khóa kéo
- Võng có mùng tẩm tồn lưu không khóa kéo
- Võng có mùng tẩm tồn lưu có khóa kéo
- Võng có mùng tẩm không có khóa kéo
- Võng có mùng tẩm có khóa kéo
- Võng có mùng tẩm tồn lưu không khóa kéo
- Võng có mùng tẩm tồn lưu có khóa kéo
- Khác

106. Mô tả mùng/võng đó để biết chính xác nó như thế nào?
Describle net/hammock your workgroup like most

---

107. Nếu không có thích nhất, thì nhóm ông/bà thích dùng loại nào khác?
If don't have which like the most, what types other can acceptable?

- ○ Mùng đơn tẩm thuốc MỀM
- ○ Mùng đơn tẩm thuốc CỨNG
- ○ Mùng đôi tẩm thuốc MỀM
- ○ Mùng đôi tẩm thuốc CỨNG
- ○ Mùng đơn tẩm thuốc tồn lưu MỀM
- ○ Mùng đơn tẩm thuốc tồn lưu CỨNG
- ○ Mùng đôi tẩm thuốc tồn lưu MỀM
- ○ Mùng đôi tẩm thuốc tồn lưu CỨNG
- ○ Võng có mùng tẩm không có khóa kéo
- ○ Võng có mùng tẩm có khóa kéo
- ○ Võng có mùng tẩm tồn lưu không khóa kéo
- ○ Võng có mùng tẩm tồn lưu có khóa kéo
- ○ Võng có mùng tẩm không có khóa kéo
- ○ Võng có mùng tẩm có khóa kéo
- ○ Võng có mùng tẩm tồn lưu không khóa kéo
- ○ Võng có mùng tẩm tồn lưu có khóa kéo
- ○ Khác

108. Mô tả mùng/võng đó để biết chính xác nó như thế nào?
Describle net/hammock your workgroup like most

---

109. Ông bà có thích loại mùng tồn lưu cứng của Chương trình Quốc gia cấp không?
Would you like the polyethylen nets provided by National Program?

- ◯ Có
- ◯ Không

110. Nếu như không thích, ông bà cho biết lý do tại sao?
If no, Why you don't like?

---

111. Ông/bà có thích loại võng có mùng mà chương trình quốc gia cấp?
Would you like the hammock nets provided by National Program?

- ◯ Có
- ◯ Không

112. Nếu như không thích, ông bà cho biết lý do tại sao?
If no, Why you don't like?

---

113. Ông/bà có loại mùng/võng nào dưới đây Ở NHÀ? Đọc đáp án
Do you have net and/or hammock at home? Reading choices

- ☐ Mùng
- ☐ Võng có mùng
- ☐ Võng không có mùng nhưng có mùng kèm theo hoặc bọc võng
- ☐ Không có cả ba

114. Ông/bà có thể mang những mùng/võng có mùng này vào RẪY không?
Can you bring nets/hamock you have from home?

- ◯ Có
- ◯ Không

115. Nếu không mang Tại sao không?
Why not - consider list

- [ ] Không có do không tiền mua
- [ ] Không có do không biết chỗ mua
- [ ] Không có do xa quá không đi được để mua
- [ ] Nhà đang dùng không thể lấy đi
- [ ] Quá nặng để mang đi
- [ ] Khác

116. NHÓM NGỦ CHUNG với ông/bà có thể mang mùng đơn và/hoặc võng có màng CÓ Ở NHÀ KHI ĐI RẪY KHÔNG? (Dự đoán)
Sleepinggroup can bring single bednet or/and hammock with net WHICH SLEEPINGGROUP HAVE AT HOME go to FARM. (approximately)

- ○ Có
- ○ Không

117. Ở trong rẫy, Ông/bà có đi lấy nước sinh hoạt/ra ngoài sau khi trời tối hoặc trước khi trời sáng không?
Is patient going to get drinking water outside location after dark or before dawn?

- ○ Có
- ○ Không

118. Ở trong rẫy, Ông/bà có đi tắm ở suối/khe nước sau khi trời tối không?
Bathing outside house after dark

- ○ Có
- ○ Không

119. Ở trong rẫy, Ông/bà còn làm gì khác nữa khi trời tối? Ghi ra (Những hoạt động bên ngoài nhà, dễ bị muỗi đốt)
Other activities at night?

120. Ở trong rẫy, Ông/bà thường bị muỗi đốt khi làm gì - kể tất cả?
*What do you do when mosquitoes bother you - all_*

- [ ] Đi tắm
- [ ] Ăn cơm
- [ ] Nấu cơm
- [ ] Đang làm việc
- [ ] Ngồi nghỉ
- [ ] Ngủ
- [ ] Khi đi từ rừng/rẫy về nhà
- [ ] Muỗi không chích tôi

121. Ở trong rẫy, Làm gì thì bị muỗi cắn nhiều nhất
What do you do when mosquitoes bother you most - Main

- ○ Đi tắm
- ○ Ăn cơm
- ○ Nấu cơm
- ○ Đang làm việc
- ○ Ngồi nghỉ
- ○ Ngủ
- ○ Khi đi từ rừng/rẫy về nhà
- ○ Muỗi không chích tôi

122. Theo đánh giá của cán bộ điều tra, có bao nhiêu màn cần được tẩm cho nhóm ngủ trong RẪY này ?
In the interviewer's judgement, how many nets need to be treated?

____

123. Theo đánh giá của cán bộ điều tra, có cần mua mùng mới không cho nhóm ngủ trong RẪY này?
In the interviewer's judgement, do new nets need to be purchased?

- ◯ Có
- ◯ Không

124. Theo đánh giá của cán bộ điều tra, loại mùng nào có giá cả hợp lý nhất và được chấp nhận nhiều nhất (số lượng và loại) cho nhóm ngủ trong RẪY này? (Bao nhiêu cái và loại nào? Thêm giá vào mỗi cái mùng)
In the interviewer's judgement, what is the most affordable, acceptable solution (Number of nets and which type)?(Add cost of each net to the list)

____

125. Theo đánh giá của cán bộ điều tra, giải pháp nào được cho là hợp lý nhất để giảm trừ lây truyền sốt rét cho nhóm ngủ trong RẪY này? (số mùng và loại mùng mà nhóm này thực sự sẽ dùng)
In the interviewer's judgement, what is the best solution to reduce malaria transmission (Number of nets and which type that people will really use)?

____

126. Theo đánh giá của điều tra viên, Nếu loại màn cần mua có giá cao, nhóm ngủ trong RẪY này có sẵn sàng chi trả 1/2 giá tiền với dự án không? (có thể hỏi người dân để trả lời câu hỏi này)
In the interviewer's judgement, If the chosen type of net has high price, can people share half cost with Project?

- ◯ Có
- ◯ Không

127. Trung bình trong một năm, có bao nhiều ngày ông/bà về nhà sau khi trời tối và trước khi trời sáng?
In average, How many days do you walk back from farm after dark before dawn?

____

. NHÓM CHỈ ĐI RẪY

128. Ông/bà có muốn tẩm hóa chất cho mùng của ông/bà (chưa được tẩm) không?

- ◯ Có
- ◯ Không

129. Ông/bà có muốn tẩm hóa chất cho cả võng và mùng của võng ông/bà (chưa được tẩm) không?
Would you be willing to treat yourentirehammockandnet

- Có
- Không

130. Nếu không tẩm cả võng và mùng của võng, thì chỉ tẩm mùng của võng được không?
Would you be willing to treat just only the net of hammock?

- Có
- Không

. CÁC HOẠT ĐỘNG VÀ MÙNG TẠI NHÀ

133. Có KHOẢNG bao nhiêu người MẮC SỐT RÉT trong bán kính 0.5 km xung quang NHÀ của ông/bà ở trong 2 năm trở lại đây?
How many people around your house in the 0.5km area GOT MALARIA in 2yrs? (APPROXIMATELY)

____

134. Khi ở NHÀ_GIA ĐÌNH của ông/bà có dùng mùng/võng HẾT CẢ ĐÊM ở mức nào? (đọc đáp án)
How often do members of your family sleep under net/hammock net in last week at home? Reading choices

- Không bao giờ
- Thỉnh thoảng
- Thường xuyên
- Luôn luôn

134a. Trong tuần qua, có bao nhiêu đêm ông/bà ngủ mà không dùng mùng? (có mấy đêm không dùng mùng)
how many nights not slept under the net in last weeks not slept under the net in last week

____

135. Có tổng cộng bao nhiêu mùng và võng TẠI NHÀ của ông/bà?
Total nets and hammocks do your family have at home?

____

136. Loại mùng và võng ở nhà ông/bà có TẠI NHÀ là gì? (CHỌN TẤT CẢ CÁC LOẠI MÀ HỌ CÓ)
Types of nets and hammocks at home (select all type they have)

- [ ] Mùng đơn
- [ ] Mùng đôi
- [ ] Mùng đơn tẩm thuốc MỀM
- [ ] Mùng đơn tẩm thuốc CỨNG
- [ ] Mùng đôi tẩm thuốc MỀM
- [ ] Mùng đôi tẩm thuốc CỨNG
- [ ] Mùng đơn tẩm thuốc tồn lưu MỀM
- [ ] Mùng đơn tẩm thuốc tồn lưu CỨNG
- [ ] Mùng đôi tẩm thuốc tồn lưu MỀM
- [ ] Mùng đôi tẩm thuốc tồn lưu CỨNG
- [ ] Võng không có mùng
- [ ] Võng có mùng không có khóa kéo
- [ ] Võng có mùng có khóa kéo
- [ ] Võng có mùng tẩm không có khóa kéo
- [ ] Võng có mùng tẩm có khóa kéo
- [ ] Võng có mùng tẩm tồn lưu không khóa kéo
- [ ] Võng có mùng tẩm tồn lưu có khóa kéo

- [ ] Võng không có mùng
- [ ] Võng có mùng không có khóa kéo
- [ ] Võng có mùng có khóa kéo
- [ ] Võng có mùng tẩm không có khóa kéo
- [ ] Võng có mùng tẩm có khóa kéo
- [ ] Võng có mùng tẩm tồn lưu không khóa kéo
- [ ] Võng có mùng tẩm tồn lưu có khóa kéo
- [ ] Khác

137. Gia đình của ông/bà có TỔNG SỐ bao nhiêu mùng TẠI NHÀ?
How many regular nets do your family have at home?

____

138. Trong đó, Có bao nhiêu cái mùng được tẩm hóa chất?
How many regular nets treated at home?

____

139. Tổng cộng bao nhiêu cái võng tại NHÀ của ông/bà?
How many hammock do YOUR FAMILY have AT HOME?

____

140. Trong đó, có bao nhiêu cái võng có mùng TẠI NHÀ của ông/bà?
How many hammock with NET do YOUR FAMILY have AT HOME?

____

141. Trong đó, có bao nhiêu cái võng mùng được tẩm hóa chất TẠI NHÀ?
how many treated hammocks do YOUR FAMILY have AT HOME?

____

142. Loại mùng/võng nhóm ông/bà thích dùng NHẤT để ngủ CẢ ĐÊM TẠI NHÀ là gì?
Type of net/hammock THIS HOUSE like most to use AT HOME?

- ◯ Mùng đơn tẩm thuốc MỀM
- ◯ Mùng đơn tẩm thuốc CỨNG
- ◯ Mùng đôi tẩm thuốc MỀM
- ◯ Mùng đôi tẩm thuốc CỨNG
- ◯ Mùng đơn tẩm thuốc tồn lưu MỀM
- ◯ Mùng đơn tẩm thuốc tồn lưu CỨNG
- ◯ Mùng đôi tẩm thuốc tồn lưu MỀM
- ◯ Mùng đôi tẩm thuốc tồn lưu CỨNG
- ◯ Võng có mùng tẩm không có khóa kéo
- ◯ Võng có mùng tẩm có khóa kéo
- ◯ Võng có mùng tẩm tồn lưu không khóa kéo
- ◯ Võng có mùng tẩm tồn lưu có khóa kéo
- ◯ Võng có mùng tẩm không có khóa kéo
- ◯ Võng có mùng tẩm có khóa kéo
- ◯ Võng có mùng tẩm tồn lưu không khóa kéo
- ◯ Võng có mùng tẩm tồn lưu có khóa kéo
- ◯ Khác

143. Mô tả mùng/võng đó để biết chính xác nó như thế nào?
Describe net/hammock THIS HOUSE like most?

---

144. Nếu không có thích nhất, thì nhóm ông/bà thích dùng loại nào khác?
If don't have which like the most, what types other can acceptable?

- ◯ Mùng đơn tẩm thuốc MỀM
- ◯ Mùng đơn tẩm thuốc CỨNG
- ◯ Mùng đôi tẩm thuốc MỀM
- ◯ Mùng đôi tẩm thuốc CỨNG
- ◯ Mùng đơn tẩm thuốc tồn lưu MỀM
- ◯ Mùng đơn tẩm thuốc tồn lưu CỨNG
- ◯ Mùng đôi tẩm thuốc tồn lưu MỀM
- ◯ Mùng đôi tẩm thuốc tồn lưu CỨNG
- ◯ Võng có mùng tẩm không có khóa kéo
- ◯ Võng có mùng tẩm có khóa kéo
- ◯ Võng có mùng tẩm tồn lưu không khóa kéo
- ◯ Võng có mùng tẩm tồn lưu có khóa kéo
- ◯ Võng có mùng tẩm không có khóa kéo
- ◯ Võng có mùng tẩm có khóa kéo
- ◯ Võng có mùng tẩm tồn lưu không khóa kéo
- ◯ Võng có mùng tẩm tồn lưu có khóa kéo
- ◯ Khác

145. Mô tả mùng/võng đó để biết chính xác nó như thế nào?
Describe net/hammock THIS HOUSE can acceptable?

146. Ông bà có thích loại mùng tồn lưu cứng của Chương trình Quốc gia cấp không?
Would you like the polyethylen nets provided by National Program?

- Có
- Không

147. Nếu như không thích, ông bà cho biết lý do tại sao?
If no, Why you don't like?

148. Ông/bà có thích loại võng có mùng mà chương trình quốc gia cấp?
Would you like the hammock nets provided by National Program?

- Có
- Không

149. Nếu như không thích, ông bà cho biết lý do tại sao?
If no, Why you don't like?

150. Trong 12 tháng qua, nhà ông/bà có được phun hóa chất bao nhiêu lần?
In the last 12 months, how many times has your house been sprayed ?

151. Trong khi phun thì khoảng bao nhiêu phần trăm tường nhà ông/bà được phun?
How many percents walls are sprayed? Approximately

152. Theo đánh giá của cán bộ điều tra, có bao nhiêu màn cần được tẩm TẠI HỘ NÀY?
In the interviewer's judgement, how many nets need to be treated for this HOUSE?

153. Theo đánh giá của cán bộ điều tra, cần mua bao nhiêu mùng mới cho TẠI HỘ NÀY?
In the interviewer's judgement, how many nets need to be purchased FOR THIS HOUSE?

154. Theo đánh giá của cán bộ điều tra, loại nào sau đây cần được mua mới?
In the interviewer's judgement, which types need to be purchased FOR THIS HOUSE?

- [ ] Mùng đơn tẩm thuốc MỀM
- [ ] Mùng đơn tẩm thuốc CỨNG
- [ ] Mùng đôi tẩm thuốc MỀM
- [ ] Mùng đôi tẩm thuốc CỨNG
- [ ] Mùng đơn tẩm thuốc tồn lưu MỀM
- [ ] Mùng đơn tẩm thuốc tồn lưu CỨNG
- [ ] Mùng đôi tẩm thuốc tồn lưu MỀM
- [ ] Mùng đôi tẩm thuốc tồn lưu CỨNG
- [ ] Võng có mùng tẩm không có khóa kéo
- [ ] Võng có mùng tẩm có khóa kéo
- [ ] Võng có mùng tẩm tồn lưu không khóa kéo
- [ ] Võng có mùng tẩm tồn lưu có khóa kéo
- [ ] Võng có mùng tẩm không có khóa kéo
- [ ] Võng có mùng tẩm có khóa kéo
- [ ] Võng có mùng tẩm tồn lưu không khóa kéo
- [ ] Võng có mùng tẩm tồn lưu có khóa kéo
- [ ] Khác

155. Theo đánh giá của cán bộ điều tra, loại màn nào có giá cả hợp lý nhất và được chấp nhận nhiều nhất (số lượng và loại) cho TẠI HỘ NÀY? (Bao nhiêu cái và loại nào? Thêm giá vào mỗi cái mùng)

In the interviewer's judgement, what is the most affordable, acceptable solution FOR THIS HOUSE?(Number of nets and which type)?(Add cost of each net to the list)

156. Theo đánh giá của cán bộ điều tra, giải pháp nào được cho là hợp lý nhất để giảm trừ lây truyền sốt rét cho TẠI HỘ NÀY? (số mùng và loại mùng mà nhóm này thực sự sẽ dùng)

In the interviewer's judgement, what is the best solution to reduce malaria transmission FOR THIS HOUSE (Number of nets and which type that people will really use)?

157. Theo đánh giá của điều tra viên, Nếu loại màn cần mua có giá cao, HỘ GIA ĐÌNH NÀYnày có sẵn sàng chi trả 1/2 giá tiền với dự án không? (có thể hỏi người dân để trả lời câu hỏi này)

In the interviewer's judgement, If the chosen type of net has high price, will THIS HOUSE be willing and able to share half cost with project?

- Có
- Không

158. Khi ở NHÀ thường mấy giờ ông/bà ngủ?

what time do you sleep AT HOME?

--:-- --

159. Ở NHÀ, Ông/bà có đi lấy nước sinh hoạt/ra ngoài sau khi trời tối hoặc trước khi trời sáng không?

Is patient going to get drinking water outside location after dark or before dawn?

- Có
- Không

160. Ở NHÀ, Ông/bà có đi tắm ở suối/khe nước sau khi trời tối không?

Bathing outside house after dark when you are at home

- Có
- Không

161. Ở NHÀ, Ông/bà còn làm gì khác nữa khi trởi tối? Ghi ra (Những hoạt động bên ngoài nhà, dễ bị muỗi đốt)

Other activities at night?

162. Ở NHÀ, Ông/bà thường bị muỗi đốt khi làm gì - kể tất cả?
*What do you do when mosquitoes bother you at home- all_*

- [ ] Đi tắm
- [ ] Ăn cơm
- [ ] Nấu cơm
- [ ] Đang làm việc
- [ ] Ngồi nghỉ
- [ ] Ngủ
- [ ] Khi đi từ rừng/rẫy về nhà
- [ ] Muỗi không chích tôi

163. Ở NHÀ, Làm gì thì bị muỗi cắn nhiều nhất
What do you do when mosquitoes bother you most at home - Main

- ○ Đi tắm
- ○ Ăn cơm
- ○ Nấu cơm
- ○ Đang làm việc
- ○ Ngồi nghỉ
- ○ Ngủ
- ○ Khi đi từ rừng/rẫy về nhà
- ○ Muỗi không chích tôi

164. Gia đình ông/bà nhận được thông tin về phòng chống sốt rét từ nguồn nào ?
What sources of information/communication on malaria have you and your family receive? (please observes and note. Multiple choice answer)

- [ ] Radio
- [ ] TV
- [ ] Tranh/báo/tạp chí
- [ ] Khác
- [ ] Không có

165. Cách nào/phương tiện nào tốt nhất để tuyên truyền/giáo dục Ông/bà về sốt rét?
Which is thebest way toeducate people about malaria

____

166. Kiểu nhà của ông/bà là gì? (Có thể quan sát và đánh vào, không cần hỏi)
Type of housing structure (observation for weath)

- ○ Nhà sàn
- ○ Nhà trệt
- ○ Khác

167. Mái nhà của ông/bà được làm bằng gì? (Có thể quan sát và đánh vào, không cần hỏi)
Roof type(observation for weath)

- ○ Tre
- ○ Gỗ
- ○ Ngói
- ○ Xi-măng
- ○ Tôn

- Lá

168. Tường nhà của ông/bà làm bằng gì? (Có thể quan sát và đánh vào, không cần hỏi)
Wall type (observation for weath)

- Gạch
- Đất
- Tre
- Lá
- Sắt
- Khác
- Không có

169. Nhà vệ sinh của gia đình ông/bà là loại gì?
Toilet type (observation for weath)

- Nhà vệ sinh tự hoại
- Nhà vệ sinh khép kín
- Nhà vệ sinh không khép kín
- Không có nhà vệ sinh
- Khác

170. Từ nhà ông/bà đến rừng bất kì bao xa? (trong rừng ghi 0)
How far is it from your house to forest?

____

171. Xung quanh nhà ông/bà có chỗ nước đọng nào không?
is there any standing water around your house?

- Có

- Không

172. Nhà ông/bà có bếp lửa giữa nhà không?
Is there any the fire in your house?

- Có
- Không

173. Ông/bà có thường tham gia họp dân/tập huấn về phòng chống sốt rét không?
How often do you come to meeting about control of Malaria in your village?

- Không bao giờ
- Thỉnh thoảng
- Thường xuyên
- Luôn luôn

173a. Ông/bà có muốn dùng kem bôi chống muỗi không?
Would you be willing to use mosquito repellant ?

- Có
- Không

173b. Ông/bà có muốn uống thuốc phòng sốt rét khi đi vào RẪY/RỪNG không? (UỐNG MỖI VIÊN MỘT NGÀY TRONG SUỐT THỜI GIAN Ở RẪY/RỪNG)
Would you be willing to take medicine before you go to the FF in the future to Anti-malaria drug? (one pill per day with meals while in forest)

- Có
- Không

174. Nguồn nước chính gia đình ông/bà sử dụng là gì TẠI NHÀ ?
main water source AT HOME?

- ○ Sông
- ○ Suối
- ○ Giếng đào
- ○ Nước mưa
- ○ Hồ/Ao
- ○ Nước giếng khoan công cộng
- ○ Nước máy
- ○ Khác

175. Nguồn nước cách rừng bao xa? (Km)
Estimate KM

____

176. Nguồn nước chính gia đình ông/bà sử dụng TẠI NHÀ trong MÙA KHÔ là gì?
main water source dry season AT HOME

- ○ Sông
- ○ Suối
- ○ Giếng/Giếng khoan
- ○ Nước mưa
- ○ Hồ/Ao
- ○ Nước giếng khoan

◯ Khác

177. Nguồn nước cách rừng bao xa? (KM)
Estimate KM

____

178. Bạn có quan tâm đến việc hướng dẫn người khác biết đến phòng và điều trị sốt rét
Are you interested in teaching others about malaria prevention and elimination?

◯ Có

◯ Không

◯ Có thể

179. Người điều tra tự đánh giá: Người này có khả năng trở thành cộng tác viên được không?
Should we consider recruiting this person as a peer educator?

◯ Có

◯ Không

◯ Có thể

180. Tổng thu nhập mỗi năm của gia đình ông/bà là bao nhiêu? (Liệt kê tổng thu nhật từ rừng, chăn nuôi, thu nhập khác... Đơn vị tính triệu đồng)
Avg income year of family

____

181. Thu nhập hằng năm từ SẢN PHẨM TỪ RỪNG HOẶC RẪY của gia đình là bao nhiêu? (Triệu đồng)
Income FOREST/FARM products

____

182. Thu nhập hằng năm từ làm việc tại RỪNG HOẶC RẪY của gia đình là bao nhiêu? (Triệu đồng) (KỂ CẢ LÀM THUÊ Ở RỪNG)
Income FOREST/FARM labor

____

183. Gia đình ông/bà có nuôi con nào dưới đây?
How many kinds of the following animals does this household own?

- [ ] Gà
- [ ] Vịt
- [ ] Bò
- [ ] Dê
- [ ] Heo
- [ ] Khác
- [ ] Không có nuôi

184. Chuồng gia xúc cách nhà ông/bà bao nhiều MÉT?
How far is it from your house to cattle cage? (meter)

____

185. Gia đình ông/bà có bao nhiêu chiếc xe máy?
How many motocycle do your family have?

____

186. Gia đình ông/bà có bao nhiêu chiếc TV?
How many television do your family have?

____

187. Gia đình ông/bà có bao nhiêu chiếc đài radio?
How many radio do your family have?

____

188. Gia đình ông/bà có bao nhiêu chiếc điện thoại di động?
How many cellphones do your family have?

____

189. Số điện thoại ông/bà? (Lưu ý ghi số điện thoại của hộ gia đình)
Phone1

---

190. Nếu không có điện thoại, xin số điện thoại người thân hoặc ở gần nhà?
Phone2

---

191. Thôn/Bản
Village village

---

192. Xã
Commune

- ◯ Xuân Quang 1
- ◯ Xuân Lãnh
- ◯ Phú Mỡ
- ◯ Thanh
- ◯ Xy
- ◯ Ba Tầng

193. Tên phỏng vấn viên
Interviewer name

---

194. Tên giám sát viên
Supervisior/Checked person name
